# Antibiotic-Free Selection in Biotherapeutics: Now and Forever

**DOI:** 10.3390/pathogens4020157

**Published:** 2015-04-03

**Authors:** Charlotte Mignon, Régis Sodoyer, Bettina Werle

**Affiliations:** Technology Research Institute Bioaster, 317 avenue Jean-Jaurés, 69007 Lyon, France; E-Mails: Charlotte.mignon@bioaster.org (C.M.); Bettina.werle@bioaster.org (B.W.)

**Keywords:** antibiotic-free selection, auxotrophy, post-seggregational killing, minicircle

## Abstract

The continuously improving sophistication of molecular engineering techniques gives access to novel classes of bio-therapeutics and new challenges for their production in full respect of the strengthening regulations. Among these biologic agents are DNA based vaccines or gene therapy products and to a lesser extent genetically engineered live vaccines or delivery vehicles. The use of antibiotic-based selection, frequently associated with genetic manipulation of microorganism is currently undergoing a profound metamorphosis with the implementation and diversification of alternative selection means. This short review will present examples of alternatives to antibiotic selection and their context of application to highlight their ineluctable invasion of the bio-therapeutic world.

## 1. Introduction

The discovery of antibiotics, considered for several decades a major step forward in the global domain of therapy against some infectious agents, is still a very active domain of investigation. Unfortunately, the excessive and sometimes inappropriate use of antibiotics is responsible for a large-scale spreading of antibiotics in the environment. The first consequence is the emergence of resistant or even multi-resistant pathogenic bacterial strains that has become a general concern promise to even further increase [1]. The improvement of existing antibiotics and discovery of new molecules is, by definition, an endless quest [2,3]. Besides therapeutic applications, antibiotics are often used as a selection pressure to avoid bio-contamination in production processes such as fermentation. In this particular context, the problem can show two distinct facets: The antibiotic molecule itself, seen as a contaminant product in a given biological and the antibiotic resistance gene used as a selection marker.

The increasing regulatory requirements to which biological agents are subjected will hopefully have a great impact in the fields of industrial protein expression and production, live vectors for bio-therapeutic agent delivery and gene therapy. There is an expectation that in the near future, the rule becomes “zero tolerance” towards antibiotic-based selection in production and delivery systems. Besides the antibiotic itself, the antibiotic resistance gene is a major subject of consideration. The complete absence of antibiotic-resistance genes being the only way to ensure that propagation in the environment or transfer of resistance to pathogenic strains will not happen. In order to address these issues, different and complementary approaches can be applied. The first would be to design more stable host/vector couples allowing to set-up and conduct fermentation processes in the complete absence of antibiotics. A more achieved strategy would be to substitute the antibiotic-based selection by an alternative mean such as the complementation of an essential gene product, not expressed by the host, poison antidote mean of selection or sophisticated post-segregational killing mechanism.

For specific therapeutic agents or fields of application such as DNA vaccination, passive immunization using antibody gene transfer [4] or gene therapy, the presence of an antibiotic resistant gene in the vector backbone is rightly pointed out as undesirable by health authorities. The major issue associated with DNA delivery or immunization being a possibility of horizontal transfer of antibiotic resistance to circulating microbial population. Such a consideration is emphasized by the very long persistence of DNA constructs upon injection, some published data indicates several months. Armengol *et al.* have used *in situ* hybridization and PCR to document persistence of a plasmid harboring the *cry11Bb* gene of *Bacillus* up to two years after injection in mouse muscle [5]. Other studies based on bio-distribution and safety of antibiotic-free plasmid encoding somatostatin genes delivered by attenuated *Salmonella enterica* serovar *Choleraesuis* revealed detection in several examined tissues with the exception of ovary and brain, but a rapid clearance from the body (approximately 10 days) [6]. The situation can be seen as worthwhile for long term and multiple delivery protocols currently used in the field of gene therapy. Finally, another domain of application must be included in the discussion, this domain being the use of live bacteria as delivery vehicles containing extra chromosomal elements that need to be stably maintained. Obviously, in that category, we will find commensal bacterial strains expressing plasmid borne foreign antigens.

### Regulatory Concern

To date, the use of the β-lactam antibiotic family is not acceptable, in order to avoid concerns for patients showing reactivity to β-lactam antibiotics. Therefore, Kanamycin and to a lesser extent, Tetracyclin, are commonly used and still acceptable to health authorities and the development of new drugs is always more challenging [7]. Susceptibility to the antibiotic compound is the visible part of the iceberg, the most critical issue being the horizontal genetic transfer of antibiotic resistant genes to prokaryotic organisms, present in the environment or in commensal flora. It becomes clear that antibiotic-resistant bacteria are causing a global health crisis and that mechanisms behind acquisition of resistance are more and more documented [8].

Horizontal genetic transfer (HGT), an important driving force in bacterial evolution, is the passage of genetic elements between organisms [9,10]. HGT is influenced by the advantage conferred by the transferred gene, the toxicity of its product, the capacity of the transferred gene to be integrated into the host genome and to be stabilized, and a certain compatibility of codon usage between the transferred gene and the host. The probability of integration and stabilization of a HGT into a new host genome is correlated and increased by the similarity between the tRNA pools of the donor and recipient organism. Acquisition of antibiotic resistance is one example of this evolution by HGT. As early as in 1965, Datta and Kontomichalou [11] showed the widespread transfer of penicillin resistance across *Enterobacteriaceae*. A marked β-lactamase activity was also measured in various *Pseudomonas aeruginosa* resistant strains in the early seventies [12]. More recently, acquisition of the virulence factors that distinguish *Salmonella* from *Escherichia coli* has been clearly shown as the result of horizontal gene transfer [13]. Recent technological breakthroughs, such as the one that can be observed for next generation sequencing, offers unthinkable and extensive possibilities of detecting numerous HGT events, especially if the data acquisition is completed by new bioinformatics tools like nucleotide substitution rate matrixes [14]. Moreover, if we consider the situation at the microbiome level, it becomes obvious that a multitude of different bacterial species, exhibiting many types of complex interactions, are likely to use HGT as a preponderant mechanism for adaptation in difficult environment [15].

For the above mentioned reasons, health agencies and global organizations (FDA, EMEA, WHO) are all going in the same direction concerning recommendations towards the limitation in use of antibiotics and propagation of antibiotics resistance genes [16]. The principal rules to be applied can be summarized as follows:

- It is strongly advised to avoid or minimize the use of any kind of antibiotics in cell or bacterial culture.
- If antibiotics are nevertheless used, it is mandatory to minimize their amount and to monitor the presence of traces in the final product.
- The rationale for the use of antibiotics must be clearly documented in the Common Technical Document (CTD).
- Penicillin, and more generally β-lactams and streptomycin, must not be used in reason of potential concerns with hyper reactivity of some patients to antibiotics of the β-lactam family
- Kanamycin and neomycin are the preferred choice and still tolerated.
- The use of antibiotic resistance markers is generally discouraged, and if used, the *in vivo* outcome and effect needs to be evaluated.

There are specific mentions for the nature of the gene encoding resistance to kanamycin, as reviewed by Williams *et al.* [7] The gene neomycin phosphotransferase III [*npt-III*, *aph(3*'*)-III*] should be avoided, since it also confers resistance to amikacin [17], a reserve antibiotic (EMEA, 2008).

As a final comment, it is easy to anticipate what the future requirements from health authorities might be: Constructs have to be completely devoid of antibiotic resistance genes in their final structure, even if in use at early stages of construction. Alternative solutions would be available and validated soon.

## 2. Vector Stabilization

Besides any consideration about pressure of selection obtained through antibiotic or antibiotic-free selection, natural stability of a given plasmid might be an additional source of investigation.

Several studies on natural plasmids, conducted in the 1970s or early 1980s, have highlighted that some plasmids naturally display regions necessary for their stability.

Plasmid F, which exists only as one to two copies per chromosome, is stably inherited to daughter cells during cell growth, contains stabilization sequences. Ogura; Hiraga [18], have shown that these sequences were independent from plasmid replication function.

Their work lead to the identification of three essential regions for plasmid maintenance:

*SopA* and *SopB* acting in *trans* and *SopC* acting in *cis* in order to stabilize the plasmid likely through interaction with, unidentified at that time, cellular components.

These authors also put in evidence that the combination *SopA*, *SopB* and *SopC* was not sufficient for a complete stabilization of mini-F plasmid, and thus identified the additional element *ccd* (control of cell death) region that seemed crucial to control cell division when copy number carrying *ccd* segment decreases [18]. The so-called *ccd* region can be split into two functional domains: *ccdB*, the product of which inhibits the host cell division and *ccdA*, able to act as an antagonist to the ccdB function. Two years after, Jaffé *et al.* [19], demonstrated that cell division is not immediately inhibited and that residual division could take place in the plasmid free-cells before finally being inhibited. Authors concluded that the *ccd* region guarantees that plasmid carrying cells could grow preferentially in a population by killing plasmid free daughter cells, introducing the concept of post-segregational killing. Plasmid R1 has also been shown to contain a stabilization system [20]. As for plasmid F, the stabilization system is based on post segregational killing due to the *parAB +* locus. This locus is composed of two genes *Hok* (Host killing) and *Sok* (suppression of killing). The translation of the *Hok* messenger, encoding a toxin lethal to the bacteria, is completely blocked by the anti-messenger *Sok*. In the absence of plasmid, *Sok*, which is less stable than *Hok*, is lost first, allowing the translation of the *Hok* mRNA and expression of the toxin lethal to the cell. Several hok mRNA paralogs have been identified in the genome of *E. coli*, and Hok protein orthologs are also found in the genomes of Enterobacteria [21,22,23].

Concerning plasmid maintenance, it has been shown that factors reducing multimerization of plasmid could increase plasmid stability [24]. ColE1 plasmid contains a region, *cer* that seems to be necessary for a recombination event converting multimers to monomers, allowing the plasmid to be more stable. Multimer resolution is achieved through action of the XerCD site-specific recombinase at the *cer* site. Precise characterization of the cer site by progressive deletions has shown the importance of its boundaries in terms of length and the apparent poorly conserved sequence among multimer resolution sites [25]. In recent work, Rcd was even more precisely described, a small regulatory RNA encoded within *cer*, which is also required. Rcd is transcribed from the P_cer_ promoter in plasmid dimers, but not in monomers, and is a key component of the checkpoint that delays the division of dimeror multimer-containing cells [26]. It was further demonstrated that a fifth chromosome-encoded protein, FIS, binds to *cer* in a sequence-specific manner. FIS appears to have little or no influence on *cer*-mediated recombination, but, along with XerC and XerD, is required for proper regulation of the P_cer_ promoter [27]. Cloning of the *cer* locus into various expression vectors has been extensively documented and the proof of principle largely established in high-cell density cultures [28].

### Multimer Resolution

The multimer resolution rule applying for plasmids has some equivalent in chromosome duplication during cell division. In Prokaryotic microorganisms, cell division can be completed only after segregation of newly replicated monomeric chromosomes. The presence of dimeric chromosomes, often due to homologous recombination between replicated chromosomes must be precisely controlled and resolved before correct completion of the cell division.

In *Escherichia coli*, dimeric chromosome segregation has been shown to be mediated by a site-specific recombination between two small sequence elements termed *dif* sites (deletion-induced filamentation). Functional *dif* site was identified as contained into a DNA fragment of 32 bp which is sufficient to allow Xer-mediated plasmid multimerization and dimer resolution. A core recombination site containing two 11 bp half-sites, flanking a 6 bp central region is the current definition of the *dif* site, and dimer resolution is achieved through action of the XerCD site-specific recombinase at the *dif* site in a similar way as *cer*.

The structural organization of *dif* is similar to that of related lambda integrase family site-specific recombination loci, e.g., *P1 loxP*, and Saccharomyces cerevisiae *FRT*.

In the context of DNA vectorization for genetic immunization or gene therapy the productivity is a major consideration and it seems useful to consider the induced metabolic stress and plasmid stability during production. Silva *et al.* have suggested possible evolution of fermentation methods towards online bioprocess monitoring to circumvent metabolic burden and plasmid instability [29]. Process optimization is a way to improve productivity, nevertheless the nature and design of the construct and recent innovations in strain and vector engineering or alterations of the host genome or vector backbone are more likely to dramatically increase production of plasmid DNA [30].

An ultimate evolution or simplification can be provided by so-called minicircle vectors that are genetic constructs devoid of plasmid bacterial sequences. The intrinsic small size of minicircles, combines safety and improved resistance to the shearing forces associated delivery methods using pneumatic devices [31].

## 3. Antibiotic-Free Selection and Plasmid Maintenance

To fulfill new regulatory constraints, to develop marker-less expression hosts or to produce safer gene delivery vectors, antibiotic-free selection systems and plasmid addiction mechanisms have been developed.

All these systems can be classified according to their mechanism of action. The different types of systems, as described in Table 1, are based on (i) complementation of auxotrophic marker, (ii) post-segregational killing (PSK), (iii) RNA-interference, (iv) de-repression of an essential gene, (v) chromosomal integration, (vi) minicircles and (vii) non antibiotic resistance.

**Table 1:** Antibiotic-free selection systems: Mechanisms of action; Applications. Abbreviations used in the table: R: Resistance; Tet: Tetracycline; Cm: Chloramphenicol; Amp: Ampicillin; Kan: Kanamycin resistance; bla: Beta-lactamase; Neo: Neomycin; Rif: Rifampicin.

Strain	Strain Modification	Plasmid Maintenance Gene	Antibiotic Resistance Gene Used for Selection	Application	Reference
**Complementation of an essential gene**					
*Escherichia coli*	*dapD* mutation → DAP auxotrohpy	*dapD*	No	DNA and protein expression	Degryse, [32]
*Salmonella typhimurium*	Δ*asd* → DAP auxotrophy	*asd*	No	Live vaccine	Galan *et al.* [33]
*Salmonella typhi*	Δ*asd* → DAP auxotrophy	*asd*	No	Vaccin delivery	Tacket *et al*. [34]
*Salmonella typhimurium and S.typhi*	*thyA* mutation → Thymidine auxotrophy	*ThyA*	No	Live vaccine	Morona *et al.* [35]
*Vibrio cholerae*	*glnA* with an internal deletion → Glutamine auxotrophy	*glnA*	No but presence of TetR and CmR genes on plasmid	Vaccine delivery	Ryan *et al*. [36]
*Salmonella enterica serovar Choleraesuis*	Δ*asd* → DAP auxotrophy	*asd*	No	Live vaccine expressing somatostatin	Liang, *et al.* [6]
*Escherichia coli*	Δ*ProBA* → Proline auxotrophy	*proBA*	Yes, CmR	protein expression	Fiedler *et al*. [37]
*Escherichia coli*	*ΔTpiA →*no growth on glycerol	*TpiA*	No but presence of AmpR and KanaR genes on plasmid	protein expression	Velur Selvamani, et al. [38]
*Escherichia coli*	*ArgE* amber mutation + *pir116 →* arginine auxotrophy	*Phe sup tRNA* + ori γ	No	DNA (pCOR)	Soubrier *et al*. [39]
*Escherichia coli*	*ThyA* amber mutation → thymidine auxotrophy	*His sup tRNA*	No	DNA (pFAR)	Marie, *et al*. [40]
*Salmonella* enteritidis	Δ*asd* → DAP auxotrophy	*asd*	No	Ghost vaccine, live vaccine	Jawale lee, [41]
*Escherichia coli*	*ΔglyA* → Glycine auxotrohpy	*glyA*		protein expression	Vidal *et al*. [42]
*Escherichia coli*	Δ*QAPRTase* → NAD auxotrophy	*QPARTase*		protein expression	Dong, et al. [43]
*Bacillus amyloliquefaciens*	Δ*gspA* → G3P auxotrophy	*glycerine-3-phosphate dehydrogenase*	No	protein expression	US2010/0248306A1
*Escherichia coli*	Δ*araD*	*araD*	No	protein expression	US2007/0036822
**Post Segregational Killing**					
*Escherichia coli*	-	hok/sok (*parB* locus)	Yes, bla	DNA and protein expression	Gerdes *et al*. [44]
*Escherichia coli*	-	hok/sok, *parDE*	Yes, KanR, AmpR	DNA and protein expression	Pecota *et al*. [45]
*Escherichia coli*	-	hok/sok (*parB* locus)		DNA and protein expression	Schweder *et al*. [46]
*Salmonella typhi*	-	hok/sok and *parA*	Yes, bla	Live vaccine	Galen *et al*. [47]
*\Escherichia coli*	-	*ccdA/ccdB*	Yes, AmpR	protein expression	Wegerer, *et al*. [48]
*Escherichia coli*	*ccdB* gene insertion	*ccdA*	No but AmpR or spectinomycinR gene present on plasmid	DNA and protein expression	Szpirer *et al*. [49].
*Escherichia coli*	*ccdB* gene insertion	*ccdA*	No	DNA and protein expression	Peubez, *et al*. [28]/WO2010007246
*Streptomyces lividans*	YoeBsl gene	YefMsl gene	No, but NeoR gene present on plasmid	protein production	Sevilliano *et al*. [50]
Chinese Ovary Hamster cell line (CHO)	Kid	Kis	No but G418R gene present on plasmid	protein production	Nehlsen, *et al.* [51]
**Repression of toxic gene**					
*Escherichia coli*	*cI-sacB*	*cI repressor*	No but CmR gene present on plasmid	DNA production	WO2010/135742
**Non-antibiotic resistance**					
*Pseudomonas putida*	Rifr Cmr hsdR1, prototrophic	tellurite resistance gene	RifR and CmR strain but no resistance on plasmid	Bioconversion of toluene	Sanchez-romero [52]
*Escherichia coli*	-	Fabl	no	DNA and protein production	Goh and Good [53]
*Pseudomonas putida/Klebsiella pneumoniae*	-	Biophalos (herbicide), mercury or arsenic resistance gene	No but KanR gene present on plasmid	Strain engineering	Herrero *et al*. [54]
**RNA-based drugless systems**					
*Escherichia coli*	*murA* under control of TetR (including a RNAI complementary seq.)	RNAi (ColE1 ori)	no	DNA or protein production	Pfaffenzeller *et al*. [55]
*Escherichia coli*	*murA* under control of TetR (including a RNAI complementary seq.)	RNAi (ColE1 ori)	no	DNA production	Mairhofer *et al*. [56]
*Escherichia coli*	RNA-IN *sacB*	RNA-OUT	no	DNA production	Luke, *et al.* [57]
**Operator Repressor Titration systems**					
*Escherichia coli*	*lac-DapD*	*lacO*	no	DNA or protein production	Nuttall, *et al.* [58], Cranenburgh, *et al.* [59]
*Salmonella enterica serovar Typhimurium*	*lac-DapD*	*lacO*	no	Live vaccine expressing *Yersinia pestis* F1 antigen	Garmory, *et al.* [60]
**Chromosomal integration (plasmid free)**					
*Bacillus subtilis*	Coat gene in fusion with heterologous peptide	No plasmid	-	Protein display on spores	Iwanicki *et al*. [61]
*Escherichia coli*	heterologous gene to be expressed	No plasmid	-	protein expression	Striedner, *et al.* [62]
*Escherichia coli*	xanthophyll biosynthetic genes from *P.nanatis* and *N. punctiforme*	No plasmid	-	protein production by metabolism modification	Lemuth, *et al.* [63]
*Escherichia coli*	GDP-l-fucose *de novo* pathway genes and *H. pylori* *futC*	No plasmid	-	protein production by metabolism modification	Baumgartner *et al*. [64]
**Minicircles**					
*Escherichia coli*	-	Amp resistance gene in producer plasmid	Yes, AmpR	DNA production for gene therapy	Chen *et al*. [65]
*Escherichia coli*	-	Amp resistance gene in producer plasmid	Yes, AmpR	DNA production for gene therapy	Chang, *et al.* [66]
*Escherichia coli*	-	Amp resistance gene in producer plasmid	Yes, AmpR	DNA production for gene therapy	Huang, *et al.* [67]
*Escherichia coli*	*ΔendA* + PhiC31 integrase gene multiple insertion	Kan resistance gene in producer plasmid	Yes, KanR	DNA production for gene therapy	Kay, *et al.* [68]
*Escherichia coli*	*ΔendA* + PhiC31 integrase gene multiple insertion	Kan resistance gene in producer plasmid	Yes, KanR	DNA production for gene therapy	Yi, *et al.* [69]
*Escherichia coli*	-	Amp resistance gene in producer plasmid	Yes, AmpR	DNA production for gene therapy	Zhang, *et al.* [70]

**Scheme 1:**
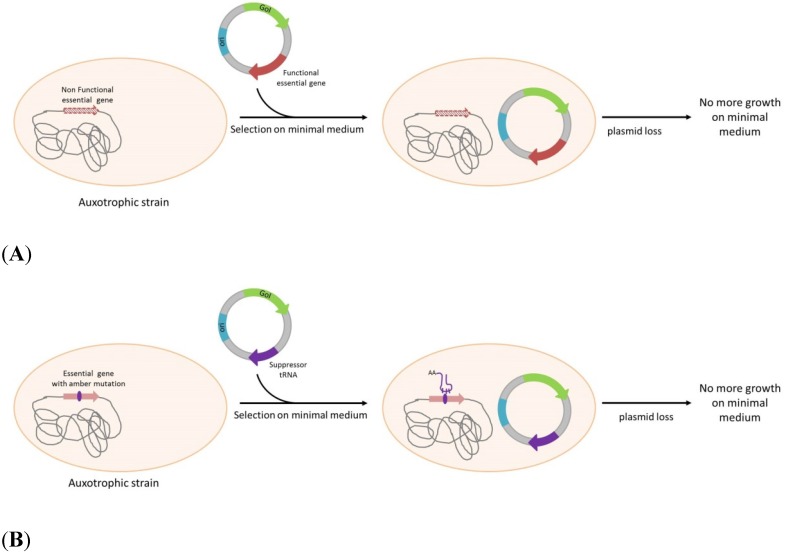
Plasmid selection and maintenance by auxotrophy complementation. (**A**): Strain auxotrophy is due to an essential gene mutation or deletion. The auxotrophy is complemented by the plasmid-born expression of the functional gene. (**B**): Strain auxotrophy is due to an amber mutation in an essential gene. Expression of the suppressor tRNA correcting amber mutation can complement the auxotrophy. GoI: Gene of Interest; ori: Origin of replication.

The first systems investigated were based on the complementation of auxotrophic markers. Among these the one established by Degryse *et al.* [32] uses an *E.coli* strain mutated in the DAP (Diaminopimelic acid) pathway due to a mutation or deletion in *DapD* gene. The plasmid carries *DapD* gene to complement the stain DAP auxotrophy. As a consequence, only bacteria containing the plasmid will grow when cultivated without DAP supplementation in the culture medium (Scheme 1A). As DAP is implicated in cell wall synthesis and mutation in the DAP pathway are lethal, several systems based on DAP complementation were investigated in other gram negative bacteria, especially *Salmonella* strains. Avirulent strains of *Salmonella* with a deletion of *asd* gene were developed to be used as live vaccines expressing a recombinant protein using a plasmid maintained with the asd complementation. Tacket *et al.* [34] constructed a *S.typhi* vaccine strain expressing the core protein of Hepatitis B virus. More recently, Liang, *et al.* [6], adopted the same strategy to express somatostatin in a *S.enterica serovar Choleraesius*. in a particular application of the same complementation, an antibiotic-free system to generate “ghost” *Salmonella* enteritidis bacteria as a vaccine. To do so, they express *PhiX174* lysis gene E in *Salmonella* enteritidis. By using another auxotrophic marker in combination with DAP auxotrophy, Wang, *et al.* [71] propose a recombinant attenuated salmonella vaccine with dual plasmid delivery system.

**Scheme 2:**
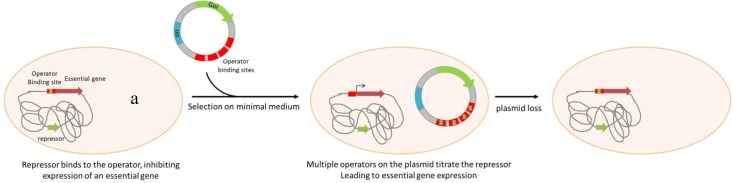
Plasmid selection and maintenance by Operator Repressor Titration. Note of a: An essential gene is repressed by a genome encoded repressor. Plasmid containing several sequences of the operator can titrate this repressor by competition, allowing essential gene expression. GoI: Gene of Interest; ori: Origin of replication.

The ORT^®^ technology (Scheme 2) uses also DAP as a central element, but in this case the regulation is based on a plasmid-mediated repressor titration driving the expression of the chromosomal *dapD* gene. The engineered host strain contains *dapD* under control of the *lac* operator/promoter *(lacO/P)*. In the absence of IPTG as inducer, cell growth is blocked by *dapD* repression. The presence, in the host, of a high copy number plasmid containing the lac operator *(lacO)* releases the repression of *dapD* expression through titration of LacI from the operator [59]. In addition to plasmid DNA production, the ORT^®^ system has been adapted for vaccine delivery using *Salmonella enterica* live bacterial vectors [72]. The system has been completed by a very elegant mean of excision of the selectable marker used for the vector cloning steps and strain engineering. The antibiotic resistance gene is flanked by dif sites that are the recognition sequence for the native Xer recombinases responsible for chromosome dimer resolution. Upon transformation and chromosomal insertion, the recombinases resolve the two directly repeated dif sites to a single site, excising the antibiotic resistance gene [73].

DAP auxotrophy and more generally the use of auxotrophic markers should have some influence on the overall productivity of a given host strain. It is nevertheless difficult to evaluate the real cost of compensating for auxotrophy [74]. The analysis would have to include the impact on specific amino acid biosynthesis pathways, the composition of the media and the additional metabolic burden associated with the expression of the plasmid-borne essential gene.

The most recently developed complementation system is based on tpiA auxotrophy in *Escherichia coli* [38]). Authors use a strain *ΔTpiA*, which presents a low growth rate with glucose as carbon source and no growth with glycerol. Only the strain carrying the plasmid expressing *tpiA* gene can restore normal growth behavior to a medium with glycerol as sole carbon source. Even if the plasmid used in the study still carries ampicillin and kanamycin resistance genes, authors show that the TpiA auxotrophy complementation is efficient enough to allow bacterial selection, plasmid stability and recombinant protein production in a 2L-batch fermentation process.

More elegant systems express a tRNA suppressor to restore an amber mutation in an essential gene. The pFAR plasmids (Marie, *et al.* [40]) allows the expression of histidine suppressor tRNA to suppress an amber mutation in the *thyA* gene. Only bacteria carrying a plasmid can grow in absence of thymidine (Scheme 1B). Plasmids called pCOR [39] use the same mechanism with a phenylalanine suppressor tRNA that suppress an amber mutation in *ArgE*. The plasmid maintenance of pCOR is improved by a conditional replication origin on the plasmid that needs *pir116*, added on the *E.coli* chromosome. Both plasmid and chromosome are in a mutually dependent, leading to a safer DNA vector that is unable to replicate in environmental bacterial strains [39].

An improved version of the system, with increased copy number, was obtained through genetic engineering in *Escherichia coli*. The bacterial gene encoding the pi initiator-protein, which plays a pivotal role in pCOR replication, was mutagenized to identify novel copy-up mutations. A particular combination of copy-up mutations was shown to produce a 3-5-fold increase in plasmid DNA per biomass unit [75].

These two systems have been mainly used for DNA vector preparation, and their applications in pre-clinical and clinical studies have been reviewed by Vandermeulen, *et al.* [76].

**Scheme 3:**
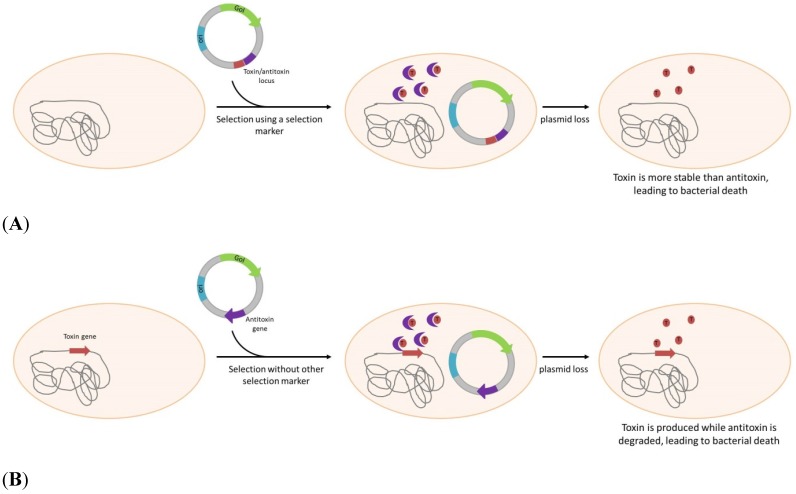
Plasmid selection or/and maintenance by post-segregational killing. (**A**): Both toxin and antitoxin genes are on the plasmid allowing plasmid maintenance. Another marker (*i.e.*, antibiotic resistant gene) is still needed to allow strain selection. (**B**): As the toxin gene is inserted into bacterial genome and antitoxin is plasmid-born, this system allows both selection and plasmid maintenance in absence of any antibiotic. GoI: Gene of Interest; ori: Origin of replication.

The second kind of system is based on a mechanism initially discovered by studying natural plasmids, called PSK. PSK consist of a plasmid locus that encodes a toxin and its own antidote, or antitoxin. In most cases, the toxin expressed is generally more stable than the antitoxin, the bacteria losing the plasmid after segregation will therefore be killed. Among the numerous toxin/antitoxin identified and recently referenced into the TADB database [77], two have been investigated for applications in biotechnology. the *parB* locus, also identified as *hok/sok*, is used for plasmid maintenance since 1986 [44] and have also been combined with *parDE* [45]. *Hok* is a messenger encoding a toxic protein, inhibited in its expression by the *Sok* anti-messenger. In case of plasmid loss, *Sok* due to shorter half-life disappears first and allow translation of the Hok protein inducing cell death (Scheme 3A). Combination of *parB* and *parA*, also showed a real improvement in plasmid maintenance [78], for a live vaccine application.

The ccdB/ccdA toxin/antitoxin pair is also used by several authors [48,79], by cloning the entire locus on the plasmid to allow its stabilization in *E.coli*. If this system is efficient for maintenance, it cannot allow a real antibiotic-free selection. For this reason, Szpirer *et al.* developed a “separate component” system where toxin is carried by the chromosome and the antidote is brought by the plasmid [49] (Scheme3B). This system, using the same genes, allows for an efficient selection of transformed bacteria. To further improve this, Peubez, *et al.* [28] proposed a way to completely eliminate the antibiotic resistant gene necessary for the construction of the vector, with a positive selection. In their strategy, the antibiotic resistant gene is flanked by both 5' and 3' ends of the *ccdA* gene containing a small homologous region. Upon transformation the two *ccdA* moieties associate through homologous recombination and give a fully functional gene, ensuring complete elimination of the antibiotic resistance gene (patent WO2010007246).

Toxin/antitoxin pairs that play an important role in plasmid maintenance and evolution in the bacterial world are numerous, and still need to be explored for future biotechnological applications [80], adjustable killing activity can even be transferred to higher eukaryotic systems [81].

Interestingly, separated components toxin/antitoxin stabilization systems have been developed for protein production in *Streptomces lividans*, using *YoeBsl* gene on the chromosome and *YefMsl* gene on the plasmid [50].

**Scheme 4:**
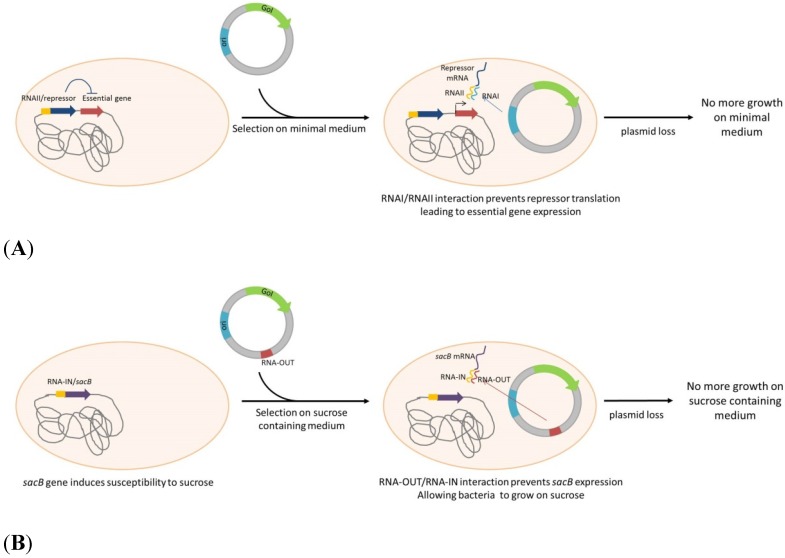
Plasmid selection and maintenance by RNA/RNA interaction. (**A**): An essential gene is under control of a repressor fused to RNAI homologous sequence. RNAI, produced from plasmid ori (ColE1 derived), binds to this homologous RNA, inhibits the repressor translation and restores the essential gene expression. (**B**): *sacB* gene is inserted into the genome, fused to RNA-IN sequence. RNA-OUT, encoded by the plasmid, binds to RNA-IN, inhibiting sacB translation, restoring ability of the strain to grow on sucrose containing medium. GoI: Gene of Interest; ori: Origin of replication.

An alternative and novel plasmid addiction model, based on ColE1 derived RNAI properties was suggested by Pfaffenzeller *et al.* [55]. The replication inhibitor RNAI of ColE1 based plasmid can serve as an antisense to bind mRNA of an engineered toxic gene, encoded by the bacterial chromosome. This system was proposed in a fine-tuned version by using the same “selection marker” serving as an antisense to alleviate the repression of an essential gene. The essential gene *murA*, implicated in cell wall synthesis, is under control of the Tet operator, repressed by the Tet repressor that integrate a complementary sequence of RNAI. In absence of plasmid, *murA* expression is repressed by *TetR*, leading to cell death (Scheme 4A). In bacteria carrying a replicating ColE1 based plasmid, RNAI interfere with its complementary sequence, repressing *TetR*, resulting in de-repression, expression of murA and cell growth [56]. By integrating other RNA/AS-RNA sequences, a system not restricted to ColE1-derived plasmids, was developed. In this system, Vectors express a RNA-OUT antisense RNA. RNA-OUT represses the expression of a chromosomally integrated, constitutively expressed counter-selectable marker (*sacB*). The expression of *sacB*, derived from *Bacillus subtilis*, induce a susceptibility to sucrose. In a culture medium containing sucrose, bacteria that lack the RNA-out plasmid will not keep growing (Scheme 4B) (Luke, *et al.* [57,82]).

The counterselectable marker *sacB* is also described in a patented but unpublished system (patent WO2010/135742). In this system, *sacB* is inserted into *E. coli* chromosome, under control of the λ *Pr* promoter. Plasmids expressing the λ *cI* 857, temperature sensitive repressor lead to the controlled inhibition of *sacB* which allows bacteria growth in presence of sucrose. The major advantage of this system is the reduction of vector size and plasmid produced can be used for protein expression as well as for DNA vector production for gene therapy.

To produce a recombinant protein, a way to avoid the antibiotic resistance marker is to integrate the gene of interest into bacterial chromosome [83]. With the development of seamless genomic integration by lambda red integrase [84], this kind of system is more and more accessible to easy establishment but cannot be considered for plasmid-based applications.

**Scheme 5:**
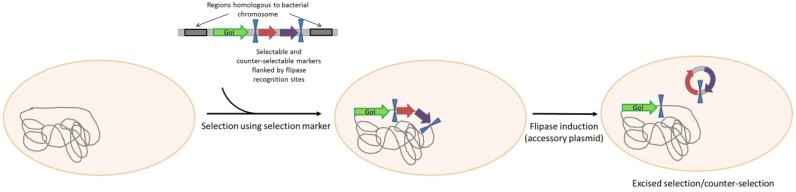
Plasmid-free expression after genomic integration. The gene of interest (GoI) is inserted into the genome to avoid antibiotic resistance gene in the production strain.

Striedner *et al.* [62] developed a plasmid-free, T7-based *E. coli* expression system in which the target gene is stably integrated into the chromosome of the host at a specific site (Scheme 5). The system, validated in real fermentation conditions, might suffer from some weaknesses due to relatively complex cloning procedure and it is also, important to consider that single copy integration might be a disadvantage for protein over-expression. One possibility to overcome the single-copy drawback might be to take advantage of a position effect, Bryant *et al.* [85] have shown that expression of gfp may vary by around 300-fold depending on its precise position on the chromosome of *E. coli* K12.

In contrast, chromosome integration is a technology of choice for some applications, such as the one described by Baumgartner *et al.* [64]. Using a chromosomal integration of recombinant genes and altering the copy number and expression of 2'-FL and intracellular GDP-L-fucose levels, the authors were able to construct and improve the first selection marker-free *E. coli* strain producing 2'-FL without the use of expression plasmids.

Another example is provided by Lemuth *et al.* [63], using a plasmid-free *Escherichia coli* strain to produce the xanthophyll astaxanthin, a high-value compound with applications in the nutraceutical, cosmetic, food, and animal feed industries.

It is also important to consider that single copy integration might be a disadvantage for protein over-expression.

**Scheme 6:**
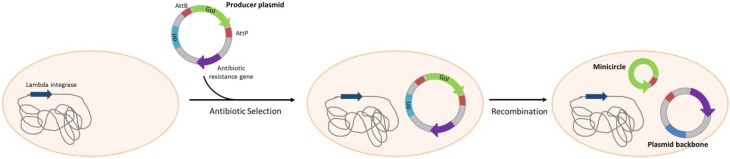
Minicircle production. The producer plasmid is selected using an antibiotic resistance gene. Cleavage and recombination leads to the formation of circular plasmid backbones and minicircles. Minicircles do not contain any antibiotic resistance gene. Strategies of *in vivo* degradation of plasmid backbone are developed to facilitate minicircle purification.

For plasmid DNA production, development of minicircle is more and more considered as an obviously interesting option. Minicircle consists of a circular DNA sequence devoid of bacterial backbone. The major advantage of these vectors is their safety due to absence of antibiotic resistance gene, bacterial replication origin and un-methylated immunogenic CpG sequence. To produce minicircle, a producer plasmid has first to be constructed. This plasmid contains a transgene expression cassette flanked by integrase or recombinase recognition sequences and all the bacterial sequences needed for selection and amplification. To propagate the producer plasmid, antibiotic selection is still used but the bioprocess is generally conducted in absence of any antibiotic to avoid minute amounts of antibiotics in the final product and to lower the process cost. After amplification of the producer plasmid, recombination at specific recognition sites lead to the production of minicircles and plasmid backbone (Scheme 6). Minicircles are more difficult to produce than conventional plasmids due to the recombination and purification steps. Minicircle generation can be done using lambda integrase [86], Cre recombinase [87] or ΦC31 integrase [65,68]. In the first production system using ΦC31 integrase, the produced plasmid contained a gene encoding homing endonuclease I-SceI and two copies of the gene encoding ΦC31 integrase and an I-SceI recognition site. ΦC31 integrase expression allows recombination of producer plasmid into minicircles and backbone plasmids. I-SceI, located in the backbone plasmid after recombination, allows the cleavage and the degradation of this plasmid. To optimize the minicircle production process, multiple copies of I-SceI and ΦC31 integrase have been integrated into the bacterial chromosome. This strategy allows for the improvement of production yields by increasing recombination efficiency and reducing contaminant backbone plasmid, facilitating minicircles’ purification.

The development of a totally antibiotic-free minicircle generation process could be considered by implementing a producer plasmid selection system using auxotrophy complementation.

Another way to avoid the antibiotic resistance is to use resistance to other chemicals. Based on this strategy, resistance to Triclosan can be provided by the expression of the plasmid-born *Fabl* gene Goh and Good [53]. Even if the chemical used is not considered as an antibiotic, this kind of antibiotic-free system presents the same limitations than antibiotic-based systems and same regulatory issues.

The toxicity of triclosan was evaluated in a rat model through exposure to aerosol inhalation [88] and its potential effect on DNA damage and reproduction was also demonstrated [89]. The results showed that the reproduction of earthworms were significantly reduced (*p* < 0.05) after exposure to triclosan concentrations ranges from 50 to 300 mg·kg^−1^. Finally, triclosan, widely used as a biocide, was also shown to induce resistance in *Aeromonas salmonicida*, a pathogen bacteria for fish [90].

Among all the systems classified in Table 1, some of them can fit two classes. For example, pFAR and pCOR plasmids, based on auxotrophic marker are categorized in Table 1 as “complementation of auxotrophic markers” but could also belong to RNA-based systems as they use t-RNA suppressor. To better understand the mechanism of action of all the antibiotic-free selection and maintenance systems, reviews including schematic have been recently published [28,76,91,92].

## 4. Gene Therapy; DNA Immunization

For different reasons, including efficiency, easier access to proof of concept and validation in the target species, DNA immunization has proven to be more successful in the domain of animal health. Various DNA vaccines for animal health have been approved.

The Fort Dodge’s West Nile DNA vaccine was approved in 2005 by the US department of agriculture. This plasmid DNA-based vaccine contains gene sequences coding WNV surface antigens prM and E within a vector background pCBWN containing an ampicillin resistance gene. The Novartis licensed DNA vaccine Apex-IHN was approved in 2005 for use in salmon in Canada to prevent from infection of hematopoetic necrosis virus (HNV). The plasmid vector expressing viral glycoprotein of HNV contains an ampicillin resistance gene. A canine melanoma vaccine (Merial, Duluth, GA, USA) was approved in the US in 2007 for the treatment of canine malignant melanoma. This vector background encodes human xenogenoic tyrosinase and contains a kanamycin resistance gene. LifeTide^®^ SW 5 is the first DNA therapy for food animals approved in Australia in 2008 (VGX animal Health, TX, USA). A swine injectable plasmid encoding porcine Growth Hormone Release Hormone carrying a kanamycin resistance gene has been approved in sows of breeding age to increase the number of piglets weaned.

Although these animal DNA vaccines are still based on antibiotic marker selection, very recently the first antibiotic free produced vaccine was approved by USDA in December 2013. The Merial’s Porcine Rotavirus vaccine based on expression of NSP4, VP4 and VP6 rotavirus antigens that prevents pigs from gastroenteritis (US 2013/0209507A1). This antibiotic free selection system uses the stabilization system StabyExpress based on the use of toxin-antitoxin genes [28,93] and anticipates future regulatory requirements.

### 4.1. DNA Vaccines for Human Application

For human applications, 365 clinical assays were initiated or completed using naked or plasmid DNA [94]. Approaches to develop antibiotic-free plasmid DNA are recommended by regulatory agencies (FDA, USDA, EMEA) for more than 15 years in order to reduce risk of allergic reactions and decrease risk of selection of antibiotic resistant pathogenic bacteria.

Although kanamycin selection marker remains allowed for gene therapy clinical trials [95,96] the first steps to increase biosafety have been performed using antibiotic-free plasmids in clinical models [76]. However, evaluation of a DNA dengue-1 vaccine in a phase I trial by an naval medical research center was still based on a VR1012 plasmid backbone containing kanamycin resistance [97].

First human phase I and II clinical trials using plasmid pCOR encoding fibroblast growth factor 1 have suggested an improvement of perfusion and an increase in amputation free survival in patients with critical limb ischemia [98]. This pCOR plasmid is an antibiotic free selection DNA vector based on complementation of amber mutation tRNA suppressor [39]. The pORT vector was chosen to develop a HIV-1 DNA vaccine, and its delivery induced a specific immune response in patients [99]. Moreover, proof of concept of gene therapy using antibiotic-free pORT plasmid AMEP coding for the recombinant desintergrin domain of ADAM 15 in disseminated melanoma has demonstrated safety and efficacy results of a phase I first-in-man study (European Society for Medical Oncology Meeting in 2012). Approval for clinical trial Phase I/II was obtained by regulatory authorities. This pORT plasmid strategy is based on “operator repressor titration” (see Table 1) [100].

A possible breakthrough technology that was not so far considered as sufficiently come to maturity, is the use of RNA-based nucleic acid vaccination [101,102]. After long and disappointing results, this technique recently entered a new era with the advent of self-replicating RNA viruses [103]. This platform has successfully entered clinical trials after encouraging pre-clinical evaluation.

### 4.2. Use of Viral Vectors

Since no DNA based vaccines for human applications received FDA approval today there is renewed interest on viral vector based vaccines. Although viral vectors have the same safety concern than nucleic DNA concerning integration capacity, they have the advantage not to contain selection markers. In animal health, ten viral vectors based vaccines are already on the market. Most of them are based on recombinant poxvirus as avian fowlpox, attenuated canarypox (Alvac), and vaccinia. The leader of the viral vector based vaccines is Merial with eight approvals based on recombinant Alvac vector, fowlpox and vaccinia. Avimex and Biomune developed fowlpox virus based and Newcastle disease virus based viral vectors respectively.

For human health, in the past years two products, Advexin (p53 based treatment of squamous cell carcinoma of the head and neck) and Cerepro (use of thymidine kinase for malignant glioma) failed the FDA and EMA approval, respectively. However, Amsterdam-based UniQure’s Glybera (alipogene tiparvovec) was approved for the treatment of Lipoprotein Lipase Deficiency (LPLD) by the European commission on September 2012. Glybera is a replication-deficient, adenovirus associated virus type 1 (AAV1) vector, containing a CMV promoter and expressing Ser447X variant of the human LPL gene [104].

In spite of a decreasing attractiveness of DNA based vaccination in human health, some developments are nevertheless in progress. One can mention the work done by Nelson *et al.* [105] on herpes simplex virus 2 with a DNA construct incorporating the regulatory agency compliant, minimal, antibiotic-free (AF) NTC8485 mammalian expression vector.

## 5. Live Vaccine; Delivery Systems

Recombinant live vaccine and delivery vectors are a specific category of bio-therapeutic agents that combine the most sophisticated technologies and complex safety/regulatory constraints. This group of biological agents is clearly distinct from old live attenuated vaccines and contains diverse technologies such as genetically attenuated pathogenic strains, GRAS [106] microorganisms (Generally Recognized As Safe) used as delivery vehicles or viral strains carrying foreign antigens.

Neglected tropical diseases, such as Chagas caused by the protozoan parasite *Trypanosoma cruzi* is a good illustration of the relevant strategy of genetically engineer a complex target for which the identification of protective antigens has proven to be difficult. The protective capacity of genetically attenuated parasite is explained by a better exposition to the different components of the immune response [107].

Lactic acid bacteria and other commensal hosts are candidates of choice for the design of novel oral vaccines and possible alternatives to attenuated pathogens [108]. A recombinant version of the extensively used BCG strain is the basis of a new strategy to improve long-lasting memory response and overcome the relative lack of efficiency of the original vaccine formula [109].

Other bacteria, such as *Samonella*, capable of activating the innate, humoral and cellular immune responses at both mucosal and systemic compartments, are therefore good vehicles to present antigens from difficult targets like HIV [110].

For all these categories of recombinant microorganisms, the requirement for antibiotic-free selection mean is absolutely essential, as they are by definition live agents prone to propagation of genetic elements.

There are numerous examples of live vaccines, for instance in the domain of animal health the MM-3 *Escherichia coli* strain a candidate for the protection of neonatal piglets against diarrhea that was originally carrying the *cat* (chloramphenicol acetyltransferase) gene. The lambda-Red technology was used to replace the first cassette by the aspartate-semialdehyde dehydrogenase gene (*asd*) and the expression levels of the two antigens K88ac fimbriae and heat-labile enterotoxin B subunit (LTB) in cell lysate were proven equivalent to MM-3 [111].

In another work based on the same kind of antibiotic-free selection, Liang *et al*. [6] evaluated the efficacy, biodistribution and safety of plasmid delivered by the strain C501. The model is based on a balanced-lethal system using an *Asd(+)* expression plasmid pVGS/2SS-asd encoding two copies of somatostatin (SS) genes carried by *Δasd/Δcrp* double mutant *Salmonella enterica* serovar *Choleraesuis*.

## 6. Conclusions

Genetic engineering technologies and regulatory requirements are continuously improving and moving in the same direction. Better techniques are creating new challenges and it is likely that in the near future the regulatory status for antibiotic-free selection will progressively move from preferred to highly recommended and mandatory. This is particularly true if we consider that credible alternatives already exist and moreover that some non-antibiotic selection means can even be advantageous options at the industrial level. Antibiotic-free selection is a field of investigation that will benefit from accumulating knowledge of biological pathways and genetic regulation of industrial microorganisms and drive an impossible step backwards.
